# A new species of *Pnigalio* (Hymenoptera, Eulophidae) parasitizing *Eriocrania semipurpurella alpina* (Lepidoptera, Eriocraniidae) in China, with its biology and a key to Chinese known species

**DOI:** 10.3897/zookeys.687.14903

**Published:** 2017-08-02

**Authors:** Tao Li, Zhong-Qi Yang, Shu-Ping Sun, Rong Wang

**Affiliations:** 1 General Station of Forest Pest Management, State Forestry Administration, Shenyang 110034, P. R. China; 2 The Key laboratory of Forest Protection of China State Forestry Administration, Research Institute of Forest Ecology, Environment and Protection, Chinese Academy of Forestry, Beijing 100091, P. R. China; 3 Qinghai Forest Pest Control and Quarantine Station, Xining 810008, P. R. China

**Keywords:** *Betula*, ectoparasitoid, *Eriocrania semipurpurella alpina*, Eulophinae, new species, *Pnigalio*.

## Abstract

A new species of Eulophinae, *Pnigalio eriocraniae* Li & Yang, **sp. n.**, is described and illustrated. This new species is a larval ectoparasitoid of *Eriocrania semipurpurella alpina* Xu (Lepidoptera, Eriocraniidae), a leaf miner in birch trees, *Betula* spp. (Betulaceae), in Qinghai Province, northwest China. The biology of the new species and a key to the known species from China are provided.

## Introduction

*Pnigalio* Schrank, 1802 (Hymenoptera: Eulophidae: Eulophinae), is comprised of 61 valid species (Noyes 2017). Eight species of *Pnigalio* were known from China (Sheng 1995; Zhu and Huang 2001, 2002; Zhang et al. 2007; Yang et al. 2015).

The species of *Pnigalio* includes numerous species which are potentially important for biological control of leaf miners belonging to Lepidoptera, Diptera, Coleoptera and Hymenoptera (Yoshimoto 1983; Askew 1984; Bernardo et al. 2006, 2007; Compton and Askew 2007; Grabenweger et al. 2009; Yefremova and Mistchenko 2009; Yegorenkova and Yefremova 2012; Strakhova et al. 2013; Yefremova et al. 2013, 2015; Noyes 2017). Four species have been reported parasitizing *Eriocrania* (Lepidoptera: Eriocraniidae) moths: *P. agraules* (Walker), *P. longulus* (Zetterstedt), *P. pectinicornis* (Linnaeus), *P. soemius* (Walker) (Askew and Shaw 1974; Hansson 1987; Koricheva 1994; Zvereva and Kozlov 2006).

*Eriocrania semipurpurella alpina* Xu has one generation a year in China. Heavy infestations in birch forests were observed in the Qilian Mountains, Qinghai Province, from 2004 to 2014. The life history and biological characteristics of *E. s. alpina* were observed (Li et al., 2016). Two ichneumonids were reported parasitizing overwintering cocoons of *E. s. alpina* (Cairangdanzhou et al., 2013; Zhang et al., 2016). A new parasitoid species of *Pnigalio* was reared from the larvae of the pest and it is described in the present paper. We also provide a key to the known Chinese species of the genus *Pnigalio*.

## Material and methods

The life history and biological characteristics of *E. s. alpina* were observed at the Beishan Forest Farm (N37°01', E102°21', 2400–2500 m), Huzhu County, Qinghai Province from 2011 to 2016. Adults of *E. s. alpina* and its parasitoids were collected using intercept traps (IT, Li et al., 2012). As well, birch leaves mined by 3rd to 4th instars larva of the pest were collected from 10 May to 16 June 2011. The leaves were dissected and examined for parasitism. Parasitoid larvae and pupae were kept in glass culture dishes (60 × 10 mm) at room temperature for rearing until parasitoid emergence. The host species was identified by Dr Hou-Hun Li (Nankai University, Tianjin, China).

For the morphological terminology used in this paper, see Bouček (1988) and Gibson (1997). The figures were taken using a Leica M205A microscope with a Leica Microsystem DFC550 digital camera. Photographs were combined using Leica Application Suite (Version 4.5.0).

The holotype, most paratypes of the new species and hosts are deposited in the Insect Museum of the General Station of Forest Pest Management (GSFPM), State Forestry Administration, Shenyang, China. Some paratypes are deposited in the Insect Museum of the Chinese Academy of Forestry (CAF), Beijing, China. Some hosts are deposited in the Insect Museum of Nankai University (NKUM), Tianjin, China.

## Taxonomy

### Pnigalio

Taxon classificationAnimaliaHymenopteraEulophidae

Schrank, 1802

Pnigalio Schrank, 1802: 315. Type-species Ichneumon pectinicornis L.

#### Diagnosis.

Body color usually metallic blue-green to blue-black (only few species black and with or without metallic reflections). Head rounded, subtriangular or subrectangular, wider than high; antenna with 2 annelli, 3–4 funicle segments and 2–3 club segments; mandible subquadrate, usually with a strongly developed acute upper tooth and 4 rounded lower teeth. Pronotum campanulate to subrectangular; scutellum with 2 or 3 pairs of bristles; propodeum with strongly developed median carina, anterior 1/3 with tongue-like projection or without projection, plicae and costulae present or absent, sometimes with additional costulae either complete or incomplete; propodeal spiracle rounded to subovate. Fore wing usually hyaline, veins developed. Metasoma elongate-ovate to narrow and long (Yoshimoto 1983).

#### Key to species of *Pnigalio* known in China

**Table:** 

1	Costulae of propodeum absent (Fig. 6), or if present, then weak and not reaching median carina (Fig. 9, arrow)	**2**
–	Costulae of propodeum present and reaching median carina	**4**
2	Axilla reticulate; fore wing length 2.3 × width, costal cell length 8.0 × width; hind leg (female) black except coxa with blue-green with purple metallic tinge	***P. scabraxillae* Yang & Yao**
–	Axilla weakly sculptured; fore wing (Fig. 11) length 2.7 × width, costal cell length 10.0 × width or fore wing length 2.1 × width, costal cell length 3.6 × width; hind femur, tibia, yellow to yellowish white (female)	**3**
3	Scape white; posterior margin of mesoscutum with three pairs of stout bristles; propodeal disc laterally reticulate; fore wing length 2.1 × width, costal cell length 3.6 × width; hind leg white, coxa smooth dorsally	***P. maijishanensis* Yang & Yao**
–	Scape blue-green with purple metallic tinge; posterior margin of mesoscutum with one pair of stout bristles (Fig. 6, arrow); propodeal disc laterally smooth (Fig. 6); fore wing (Fig. 11) length 2.7 × width, costal cell length 10.0 × width; hind femur, tibia (apical portion brown), yellow to yellowish white, coxa with coarse reticulate sculpture dorsally	***P. eriocraniae* Li &Yang, sp. n.**
4	Costula meeting anterior margin of propodeum or anterior part of median carina	**5**
–	Costula meeting median portion of median carina	**6**
5	Costula reaching anterior margin of propodeum or anterior part of median carina; mesoscutum with micro-reticulate sculpture	***P. longulus* (Zetterstedt)**
–	Costula reaching anterior 2/5 of median carina; mesoscutum with reticulate	***P. flavipes* (Ashmead)**
6	Gaster of female 1.4–1.8 times as long as broad, usually shorter than mesosoma	**7**
–	Gaster of female twice as long as broad, longer than mesosoma	**8**
7	Hind tarsus of female with all segments from pale testaceous to fuscous, never white; inner face of mid-coxa with some setae	***P. soemius* (Walker)**
–	Hind tarsus of female with one to three basal segments whitish; inner face of mid-coxa without setae	***P. agraules* (Walker)**
8	Legs of female with femora and tibiae predominantly pale yellow, only slightly fuscous; in male the dark coloration is more extensive; mesosoma green; wings hyaline	***P. phragmitis* (Erdös)**
–	Legs of female reddish-testaceous, blackish, or often a combination of the two colors, never pale yellow; mesosoma bronze-green or blue-green to almost black; fore wing of female usually with yellowish or greyish tinge	***P. pectinicornis* (L.)**

### Pnigalio eriocraniae

Taxon classificationAnimaliaHymenopteraEulophidae

Li & Yang sp. n.

http://zoobank.org/22A447DD-FD41-47E4-AAA1-245E6DB6C4A4

Figures 1 , 2 , 3–10 , 11 , 12–17

#### Etymology.

The specific name is derived from the host’s generic name *Eriocrania*.

#### Type material.

Holotype, ♀, (GSFPM), Ganchonggou, Huzhu County, Qinghai Province, leg. Tao Li, 31 May 2011, collected using intercept traps. Paratypes (101♀♀ 60♂♂): (GSFPM, 36♀♀ 12♂♂ are deposited in CAF): 32♀♀ 2♂♂, same data as holotype; 1♀ 4♂♂, same but 10 May 2011; 13♀♀ 15♂♂, same, 30 May 2011; 9♀♀ 5♂♂, same, 1 June 2011; 5♀♀ 9♂♂, same, 2 June 2011; 5♀♀ 3♂♂ (reared from larva of *Eriocrania semipurpurella alpina* Xu), same, 27 June 2011; 11♀♀ 6♂♂ (reared), same, 28 June 2011; 7♀♀ 4♂♂(reared), same, 29 June 2011; 18♀♀ 12♂♂ (reared), same, 30 June 2011.

#### Diagnosis.

**Figure 1.**
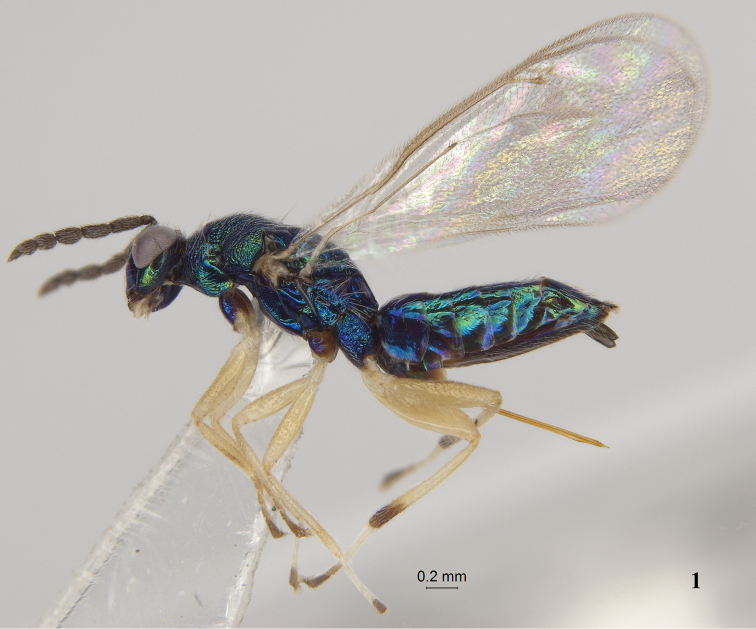
*Pnigalio eriocraniae* Li & Yang, sp. n., female, holotype, habitus lateral.

Body (Fig. 1) green to blue-green with purple metallic tinge. Antenna (Fig. 5) dark brown. Scape same color as body. Posterior margin of mesoscutum with one pair of bristles (Fig. 6, arrows). Propodeal disc smooth; costulae absent (Fig. 6) or if present, then weak and not reaching median carina (Fig. 9, arrow). Fore wing (Fig. 11) length 2.7 × width; costal cell length 10.0 × width. Hind coxa (Fig. 7) coarse reticulate dorsally; hind femur, tibia (apical portion brown), yellow to yellowish white.

#### Description.

*Female*, holotype (Fig. 1). Length of body of females 3.1–3.4 mm. and of fore wing 2.8–3.0 mm. Body green to blue-green with purple metallic tinge. Vertex golden-green. Antenna (Fig. 5) dark brown. Mandible brown. Maxillary and labial palpi, tegula, fore leg (coxa same color as body, tarsi and claw pale brown), mid leg (coxa same color as body, tarsus 4 brown), hind leg (coxa same color as body, apical portion of tibia and tarsi 3–4 brown) yellow to yellowish white. Wing membrane hyaline, venation and pilosity brown.

*Head.* In dorsal view, width 2.8 × length. Ocellar triangle convex, micro-reticulate, smooth with long brown setae. Ocelli medium-sized, and lateral areas of ocellar triangle concave. POL 1.7 × OOL, OOL 1.6 × OD. Area between eyes and ocellar triangle smooth. Head (Fig. 3) in anterior view width 1.4 × height. Eye oval, with dense microtrichia; length 1.3 × width. Malar space 0.5 × length of eye, malar sulcus straight and obvious. Face (Fig. 3) smooth, micro-reticulate texture, with sparse long white setae; Median portion of lower face with fine transverse wrinkles. Lower margin of toruli located above ventral line of eyes (Fig. 3). Distance between toruli 0.9 × diameter of toruli, 0.7 × distance between socket and eye. Antenna (Fig. 5) with 4 funiculars and 2 clavomeres. Scape length 3.8 × its width, reaching median ocellus, 3.3 × as long as pedicel. Pedicel length 1.4 × its width. Funicle 1 length 2.8 × as long as pedicel. Ratio of length of funicles 1.6:1.4:1.3:1.0, and ratio of width 1.0:1.1:1.1:1.1. Clavomere 1 length 1.3 × as long as segment 2.

**Figure 2.**
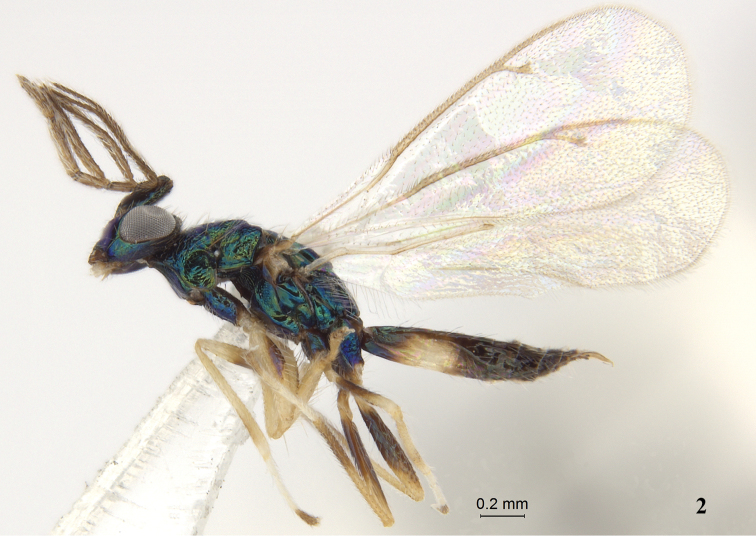
*Pnigalio eriocraniae* Li & Yang, sp. n., male, paratype, habitus lateral.

*Mesosoma* (Figs 6–7). Width about 1.2 × as long as head. Mesosoma reticulate, length 1.4 × width. Pronotum length 0.3 × as long as mesoscutum, width 0.7 × mesoscutum. Mesoscutum (Fig. 6) slightly convex, length 0.7 × its width, with dense white setae; anterior half of notaulus obvious; median and apical portions of mesoscutum reticulate, setae relatively sparse; posterior margin with one pair of stout bristles (Fig. 6, arrows). Axilla elongate, micro-reticulate. Mesoscutellum (Fig. 6) nearly circular, sublaterally more coarsely reticulate than apical and median portions; laterally micro-reticulate; with two pairs of bristles. Dorsellum narrow, median length 0.6 × as long as propodeum length. Propodeal disc (Figs 6, 9) smooth; width of median area 1.6 × its length; costula incomplete (Fig. 9, arrow); spiracles nearly circular, posterior to hind margin of metanotum; callus densely setose. Fore wing (Fig. 11) length 2.7 × width; costal cell length 10.0 × width; area of speculum mostly bare posterior to parastigma; marginal vein length 1.3 × length of submarginal vein, 1.8 × length of postmarginal vein; postmarginal vein length 2.1 × length of stigma. Lateral and ventral panel of pronotum and prepectus with coarse reticulate sculpture; mesepisternum (Fig. 7) imbricate anteriorly; subalar area and upper mesepimeron smooth. Dorsal area of hind coxa (Fig. 7) reticulate; basitarsus (Fig. 8) length 0.8 × as long as tarsus 2.

*Metasoma* (Fig. 10). Elongate-ovate in dorsal view; length about equal to head plus mesosoma, 2.0 × width of metasoma. Tergite 1 smooth; lateral area of tergite 2 with sparse white setae; sub-lateral portion of tergite 3 with sparse white setae; tergites 4–7 with dense setae; ratio of length of tergites 7.0:2.5:3.0:3.5:4.0:2.0. Ovipositor sheath slightly longer than apex of metasoma.

**Figures 3–10.**
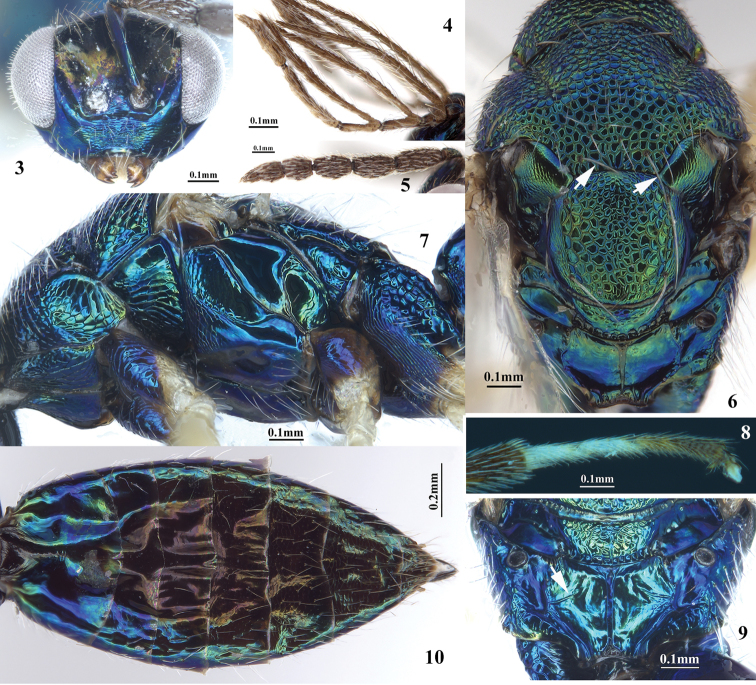
*Pnigalio eriocraniae* Li & Yang, sp. n., female (**3, 5–10**) male (**4**). **3** Head, anterior view **4** Antennal flagellum **5** Antenna **6** Mesosoma, dorsal view (bristle, arrow) **7** Mesosoma, lateral view **8** Hind tarsi **9** Propodeum (costula, arrow) **10** Metasoma, dorsal view.

*Male* (Figs 2, 17). Length of body 2.1–2.6 mm, and of fore wing 2.1–2.3 mm. Similar to female except as follows: Antennal (Fig. 4) flagellum dark brown; fore leg with coxa same color as body, basal half of femur brown with purple metallic tinge, apical tarsomere brown; mid leg with coxa same color as body, most of femur brown with purple metallic tinge, tarsus 4 brown); hind leg with coxa, most of femur same color as body, trochanters, apical half of tibia, tarsus 4 brown to fuscous, apex of femur and basal half of tibia yellowish brown; apex of tergite 1, tergite 2 and basal half of tergite 3 yellowish white to yellowish brown. Scape length 3.2 × width, 4.7 × length of pedicel; pedicle nearly circular; ratio of length of funiculars (Fig. 4) 1.0:1.6:1.6:3.1; funiculars 1–3 pectinate, projections with long setae. Dorsellum smooth, micro-reticulate. Costula absent.

#### Variation.

The variation of specimens is mainly focus on the body color, size, and costulae absent or present. The body color green with metallic tinge (26♀♀ 13♂♂) to blue-green with purple metallic tinge (76♀♀ 47♂♂); tarsi 1–3 of fore leg yellowish (72♀♀, others pale brown); apical portion of mid tibia brown (68♀♀); costula weak (79♀♀, Fig. 9) or absent (23♀♀ 60♂♂, Fig. 6). Costulae of male absent.

#### Biology.

Parasitoid eggs were deposited on the surface of the host’s cuticle (Fig. 12). It is a larval ectoparasitoid (Fig. 13) of the third to fourth instar larvae of *E. s. alpina* Xu (Lepidoptera, Eriocraniidae) which forms leaf mines on birch trees, *Betula platyphylla* Suk., *B. albo-sinensis* Burkill and *B. utilis* D. Don (Betulaceae) in Qinghai Province.

**Figure 11.**
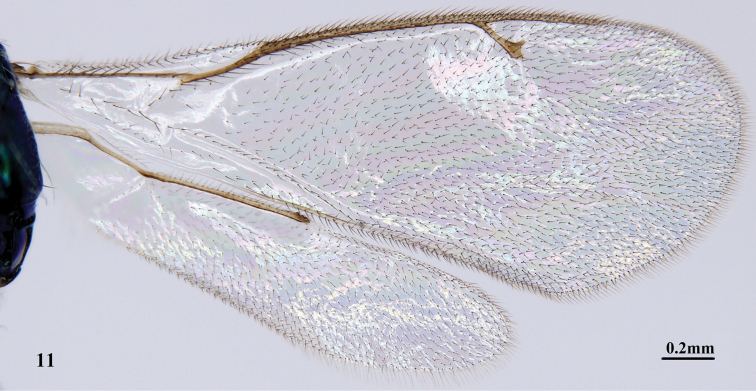
*Pnigalio eriocraniae* Li & Yang, sp. n., female, holotype, wings.

The prepupa (Fig. 14) is motionless, fusiform and with distinct lateral protuberances along the abdominal segments, length of body about 0.6–0.7 mm. The pupa is initially white to white brown (Fig. 15) and then begins to darken to brown or black (Fig. 16), with length 0.4–0.5 mm.

#### Distribution.

Northwestern China (Qinghai Province)

#### Remarks.

The new species is similar to *Pnigalio maijishanensis* Yang & Yao but can be distinguished from the latter by the following combination of characters: scape blue-green with purple metallic tinge; propodeal disc laterally smooth; hind coxa with coarse reticulate sculpture dorsally; hind femur, tibia (apical portion brown), yellow to yellowish white. In addition, the shape of the costulae and the stout bristle are different as indicated in the key.

**Figures 12–17.**
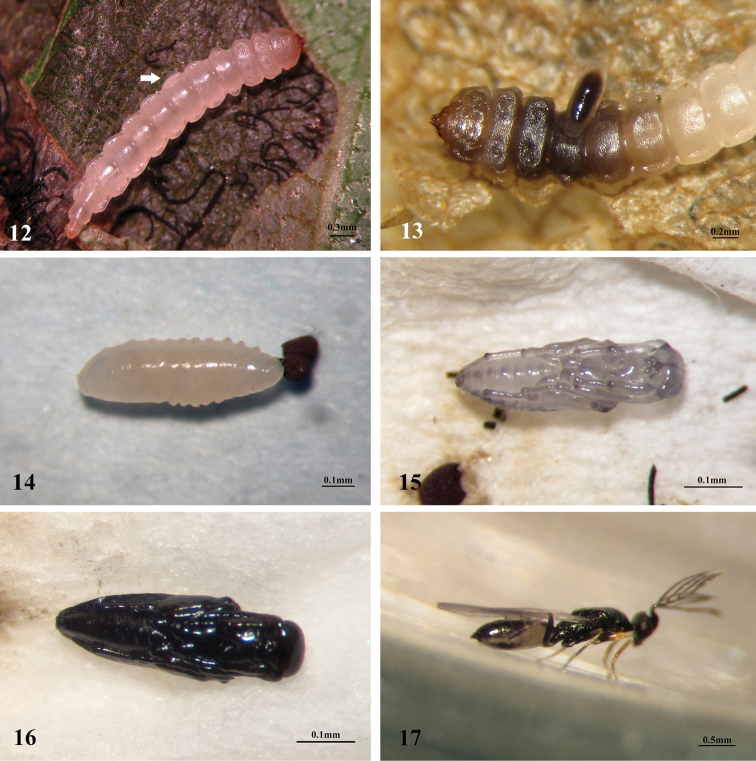
The preimaginal stages of *Pnigalio eriocraniae* Li & Yang, sp. n. **12** Egg on 4th instar larva of *E. semipurpurella alpina*, arrow **13** Larva of *P. eriocraniae* parasitizing 4th instar larva of *E. s. alpina* **14** Prepupa **15** Early pupa **16** Mature pupa **17** Emerged male.

## Supplementary Material

XML Treatment for Pnigalio

XML Treatment for Pnigalio eriocraniae
